# Social resource foraging is guided by the principles of the Marginal Value Theorem

**DOI:** 10.1038/s41598-017-11763-3

**Published:** 2017-09-12

**Authors:** Courtney Turrin, Nicholas A. Fagan, Olga Dal Monte, Steve W. C. Chang

**Affiliations:** 1 Department of Psychology, Yale University, New Haven, CT 06511 USA; 2 Department of Neuroscience, Yale School of Medicine, New Haven, CT 06520 USA

## Abstract

Optimality principles guide how animals adapt to changing environments. During foraging for nonsocial resources such as food and water, species across taxa obey a strategy that maximizes resource harvest rate. However, it remains unknown whether foraging for social resources also obeys such a strategic principle. We investigated how primates forage for social information conveyed by conspecific facial expressions using the framework of optimal foraging theory. We found that the canonical principle of Marginal Value Theorem (MVT) also applies to social resources. Consistent with MVT, rhesus macaques (*Macaca mulatta*) spent more time foraging for social information when alternative sources of information were farther away compared to when they were closer by. A comparison of four models of patch-leaving behavior confirmed that the MVT framework provided the best fit to the observed foraging behavior. This analysis further demonstrated that patch-leaving decisions were not driven simply by the declining value of the images in the patch, but instead were dependent upon both the instantaneous social value intake rate and current time in the patch.

## Introduction

Optimal foraging theory describes the behavior of animals seeking out resources in a patchy environment according to an energy-maximizing strategy. As animals use the supply of resources within a patch, patch value naturally declines and animals must decide when to leave the current patch in search of a new one. The Marginal Value Theorem (MVT) is a broadly applied optimality model that predicts foraging behavior in a variety of taxa^1–3^. MVT predicts that animals should leave the current patch when the energy intake rate within the patch diminishes to the average energy-harvesting rate in the environment^4,5^. Thus, the time that animals spend within a patch (i.e., patch-residence time) depends upon a variety of factors, including the value of the current patch (in terms of the resource being consumed), the value of other patches in the environment, and the time it would take to travel to the next closest patch (i.e., travel time).

The optimality of the strategies animals use to forage for primary resources such as food and water has been studied broadly. Across taxa, animals seek out primary resources in accordance with MVT, spending relatively more time in high quality patches that are farther from other patches in the environment and less time in patches that are low quality and nearer to other patches^1,3,6–8^. This optimal foraging model has also been applied to describe foraging for nonsocial information. Human subjects foraged for scholarly publications^9^; written content in online web searches (e.g., ref.^10^); and even their own memories^11^. These studies used a patch foraging framework to model humans’ strategic decision processes focused on exploiting nonsocial information. Although information is inherently difficult to quantify relative to primary resources, individuals’ nonsocial information-foraging decisions could be described using an optimal foraging model^9–11^.

It remains unknown whether a general foraging strategy (as described in refs^9–11^) can be applied to information seeking in the *social* domain, and more specifically, to extracting social information from others. Importantly, compared to explicitly written information, social information conveyed by others is more derivative and subjective. When foraging for social information, animals are not only extracting information from the environment, but also from conspecifics in a manner that is inherently dynamic and contingent upon social interactions. Information from conspecifics may have immediate repercussions for survival, affiliative relationships, and reproduction, among other critical factors. An adaptive strategy to maximize social information gathered during inter-individual encounters could improve an animal’s social competency and reproductive fitness.

We were interested in whether MVT could be applied to describe social animals’ foraging strategy for regulating the intake rate of social information. In this study, we used a simplified form of social information, novel images of unknown conspecific faces. Importantly, these images did not reduce subjects’ uncertainty about their environments, as information in Shannon’s classical definition should^12^; however, they did contain details about the pictured individual’s sex, age, and emotional state, which the viewer could extract by looking at the image. Thus, where we refer to social information, we are referring to this simplified definition and not Shannon’s classical definition.

We investigated social information foraging in male rhesus macaques (*Macaca mulatta*). Rhesus macaques live in large social groups. In the wild, females remain within their natal group and inherit rank from their maternal line^13^. In contrast, males disperse prior to reaching sexual maturity, either alone or with other males from their natal group^14,15^. Since males encounter novel conspecifics during the dispersal period and constantly fight to achieve and maintain their hierarchical position, effective interpretation of social information from conspecifics is especially critical to their survival.

**Figure 1:**
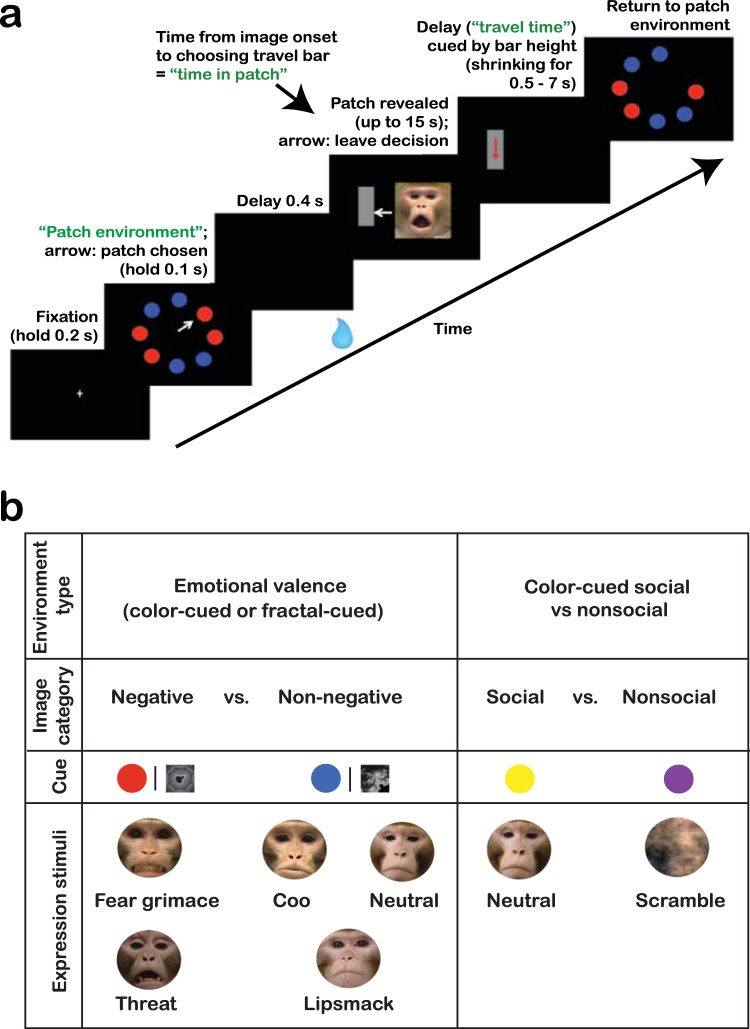
Social Information Foraging Task. (**a**) Eight targets (“patches”) are displayed initially. Target color indicates environment and image type (e.g., *color-cued emotional valence environment*: red cues a negative-valence image, blue cues a non-negative image). After patch selection, the monkey receives a standard-size reward during a 0.4-s delay, regardless of patch type chosen. A random image of the chosen valence is displayed for up to 15s. At any time, the monkey may select the “travel bar” to leave the patch and return to the remaining patches. The vertical height of the travel bar corresponds to the time to return to the patch environment (“travel time”, randomized across patches). (**b**) Subjects explored three types of environments. In the *color-cued emotional valence* and *fractal-cued emotional valence environments*, monkeys were presented with patches corresponding to images of negative (threat, fear) and non-negative (coo, lipsmack, neutral) facial expressions. These environments differed only in the cues corresponding to these image categories (red and blue targets for *color-cued*, and two black-and-white fractals for *fractal-cued*). The third environment type was the *color-cued social vs nonsocial*, in which patches corresponded to images of neutral facial expressions or scrambled faces, which were cued by yellow and purple targets, respectively.

In each block of our Social Information Foraging Task, monkeys were presented with two target types, corresponding to two categories of conspecific facial expression (Fig. 1a,b). Upon selection of a target, a single face was presented. The subject could return to target selection at any time during the face presentation by selecting a “travel bar” target, which was presented alongside the face. Described in terms of MVT, our block types were “patch environments” and the targets represented “patches” of social information. The “travel time”, which is the time from leaving the current patch to entering the next patch, was indicated by the height of the rectangular travel bar target that monkeys could select to leave the patch at any time. If monkeys obey MVT when foraging for social information, they should attempt to maximize the social information harvest rate within the explored environments.

Assigning value to social information is challenging. The value of food resources can be assessed according to caloric content^1^, and the value of liquid resources can be assessed by volume^2^, but social value is less easily quantified and thus more subjective. Here we used looking behavior to approximate the value of social images, as these measures have been shown to correlate with reinforcing value of social stimuli^16,17^. Using these estimates, we simulated optimal social information foraging based on MVT. We then compared the fits of four models of patch-leaving probability to assess the validity of using an MVT-based approach. Our MVT-based model (Eq. (4)) included terms for current social value of the patch *r*(t) and current time in the patch *t*. To determine whether including both of these terms improved model fit, we compared this model to a second variation that included the time terms but not the value term (Eq. (5)), and a third variation that included the value term and not the time term (Eq. (6)). Finally, we fitted our data to another model commonly tested within foraging frameworks, hyperbolic delay-discounting (e.g., refs^2,18–20^), in which the ‘value’ of the resource in the patch is inversely scaled by the delay required to receive it (Eq. (7)).

We hypothesized that (1) residence time within patches would increase with travel delay time, as predicted by MVT; and (2) an MVT-based model including both *t* and *r*(t) terms would outperform other models in predicting patch-leaving behavior.

## Results

In our Social Information Foraging Task, monkeys foraged in three environment types (1): *color-cued emotional valence* (2), *fractal-cued emotional valence*, and (3) *color-cued social vs nonsocial* (Fig. 1b). In both emotional valence environments, negative patches contained images of negative-valence expressions (threat, fear) and non-negative patches contained images of non-negative expressions (coo, lipsmack, neutral). In the first environment, red and blue targets cued negative and non-negative facial expressions, respectively, whereas in the second environment black-and-white fractal patterns were used to cue negative and non-negative facial expressions. This was done to ensure that preferences were based on image valence rather than target color. In the *color-cued social vs nonsocial environment*, we assessed the baseline social preference in the absence of explicit emotional valence. Social patches contained images of conspecifics with neutral facial expression, and nonsocial patches contained scrambled versions of these images.

### Patch preference

**Figure 2:**
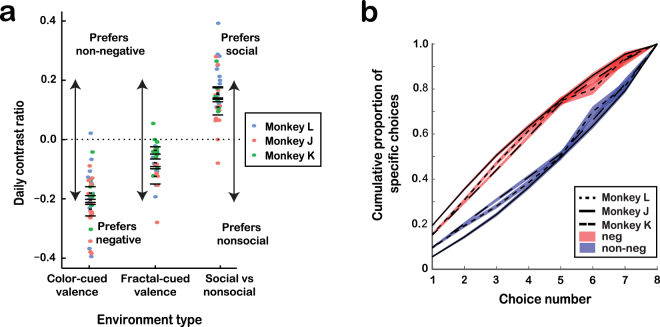
Behavioral patterns of social information foraging. (**a**) Daily contrast ratios (CR) indicating preference in *color-cued emotional valence*, *fractal-cued emotional valence*, and *color-cued social vs nonsocial environments* (see Methods). Narrow horizontal black lines indicate mean daily CRs (within-subject) and wide horizontal lines indicate standard errors. (**b**) Patch choice order within the *color-cued emotional valence environment* presented as the cumulative proportion of negative-valence (red) or non-negative (blue) patches. Black lines indicate the means (within-subject) and shading indicates standard errors.

We first demonstrate that there were social valuation processes guiding foraging behaviors (Fig. 2a). Monkeys consistently chose more negative than non-negative patches (Monkey L: *t*_13_ = −6.514, *P* < 0.0001; Monkey J: *t*_14_ = −10.309, *P* < 0.0001; Monkey K: *t*_9_ = −8.441, *P* < 0.0001, two-tailed *t*-test; Fig. 2a). Moreover, their preference was evident from their first choice within each new environment (L: χ^2^_1_ = 31.704, *P* < 0.0001; J: χ^2^_1_ = 227.071, *P* < 0.0001; K: χ^2^_1_ = 42.354, *P* < 0.0001; χ^2^ test). Across all *color-cued emotional valence environments*, the proportion of negative patches that monkeys chose showed a significant upward shift in the distribution compared to the proportion of non-negative patches chosen (Fig. 2b; L: *D* = 0.126, *P* < 0.0001; J: *D* = 0.118, *P* < 0.0001; K: *D* = 0.121, *P* < 0.0001, Kolmogorov–Smirnov test), demonstrating that monkeys sequentially explored negative patches before non-negative patches within a given environment. Interestingly, though all subjects preferred to visit negative over non-negative patches, the average looking-time durations at the images of negative and non-negative conspecific expressions were comparable (negative: 1277 ± 22 ms [mean ± standard error]; non-negative: 1321 ± 16 ms; all monkeys, *P* = 0.139, Wilcoxon test).

To confirm that patch preferences were based on emotional valences rather than the color used to cue the patch type, we tested our subjects on a *fractal-cued emotional valence environment*, where both fractal images were black-and-white. The preference for negative-valence images observed in the *color-cued valence environment* remained in the *fractal-cued valence environment* (L: *t*_6_ = −4.764, *P* < 0.01; J: *t*_7_ = −4.837, *P* < 0.01; K: *t*_12_ = −2.982, *P* = 0.011, two-tailed *t*-test; Fig. 2a). The weaker preference observed in the *fractal-cued valence environment*, albeit clearly significant, is likely due to the stimuli, two black-and-white fractals, being less saliently distinct than the stimuli used in the *color-cued valence environment* (red vs. blue targets). We tested the preference for social images (neutral facial expressions) over nonsocial images (their scrambled counterparts) in a *color-cued social vs nonsocial environment*. Monkeys preferred social over nonsocial images (L: *t*_11_ = 8.596, *P* < 0.0001; J: *t*_12_ = 4.153, *P* < 0.01; K: *t*_5_ = 6.087, *P* < 0.01, two-tailed *t*-test; Fig. 2a), and this preference was also evident from the first patch chosen within each new environment (L: χ^2^_1_ = 38.859, *P* < 0.0001; J: χ^2^_1_ = 29.137, *P* < 0.0001; K: χ^2^_1_ = 32.278, *P* < 0.0001; χ^2^ test). Taken together, monkeys preferred to forage for negative-valence facial expressions and for social over nonsocial stimuli.

### Test of MVT

**Figure 3:**
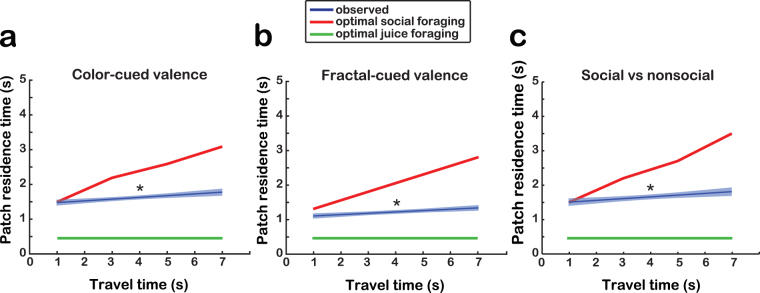
Relationship between residence time in a patch and travel time to the next patch. (**a**) Optimal and observed patch-residence times (0–5 s) in *color-cued emotional valence environment* trials as a function of randomized travel time (1–7 s). Optimal juice (green) and social information (red) foraging curves were simulated according to the adapted MVT model that included both time and reward terms (see Methods). Regression (±standard error) of observed patch-residence times are indicated in blue. (**b**) Optimal and observed patch-residence time in *fractal-cued emotional valence environment* trials. (**c**) Optimal and observed patch-residence times in *color-cued social vs nonsocial environment* trials.

A key prediction of MVT is the positively correlated relationship between patch-residence time and travel time to the next patch for generating patch-leaving decisions^5^. Examination of patch-residence time in the *color-cued valence environment* revealed a significant positive relationship with travel time (all monkeys: τ = 0.069, *P* < 0.0001, *n* = 3187 patch-leaving decisions, slope = 0.050, *P* < 0.0001, one-tailed Kendall’s correlation test; Fig. 3a). This relationship remained robust in individual monkeys’ foraging behaviors (Monkey L: τ = 0.044, *P* = 0.030, *n* = 1004; Monkey J: τ = 0.040, *P* = 0.013, *n* = 1541; Monkey K: τ = 0.047, *P* = 0.021, *n* = 1061). A similar positive relationship between patch-residence time and travel time was observed across both patch types in the *color-cued valence environment* (negative patches: τ = 0.066, *P* < 0.0001, *n* = 1923 patch-leaving decisions, one-tailed Kendall’s correlation test; non-negative patches: τ = 0.074, *P* < 0.001, *n* = 1264). These findings were replicated by the results from the *fractal-cued valence environment* (all monkeys: τ = 0.079, *P* < 0.0001, *n* = 3945 patch-leaving decisions, slope = 0.039, *P* < 0.0001, one-tailed Kendall’s correlation test; negative patches: τ = 0.066, *P* < 0.0001, *n* = 2083; non-negative patches: τ = 0.094, *P* < 0.0001, *n* = 1862; Monkey L: τ = 0.061, *P* < 0.001, *n* = 1875; Monkey J: τ = 0.092, *P* < 0.0001, *n* = 1050; Monkey K: τ = 0.073, *P* < 0.001, *n* = 1397; Fig. 3b). Furthermore, patch-leaving behavior in the *color-cued social vs nonsocial environment* was also positively correlated with travel time, indicating a similar foraging strategy when monkeys foraged for neutral expression or scrambled faces (all monkeys: τ = 0.075, *P* < 0.0001, *n* = 2142, slope = 0.051, *P* < 0.0001; social patches: τ = 0.083, *P* < 0.0001, *n* = 1201; nonsocial patches: τ = 0.063, *P* = 0.004, *n* = 941; Monkey L: τ = 0.040, *P* = 0.056, *n* = 897; Monkey J: τ = 0.041, *P* = 0.058, *n* = 732; Monkey K: τ = 0. 124, *P* < 0.0001, *n* = 685; Fig. 3c). Therefore, all monkeys tested showed foraging patterns that were consistent with MVT across all foraging environments and all patch types.

**Figure 4:**
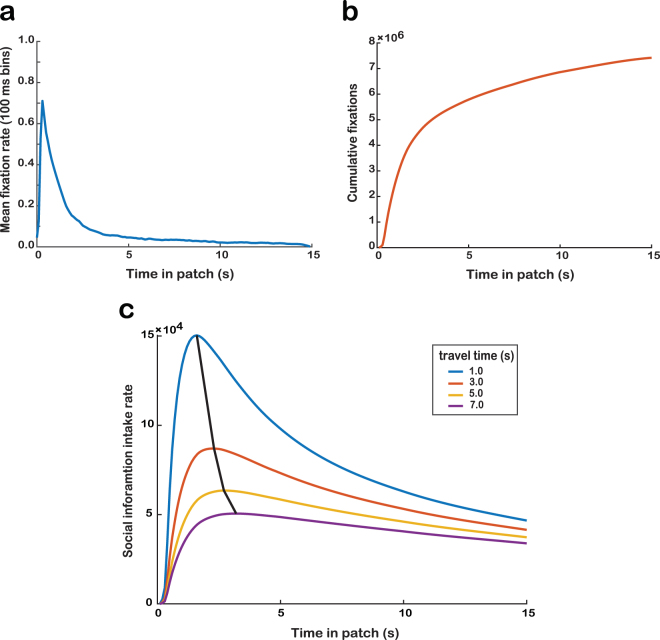
Simulation of optimal social foraging strategy based on MVT. (**a**) Mean rate of fixation to the image in the 15-s image-viewing period across all trials (see Methods) (**b**) Social information intake function *g*(t) based on looking behavior. Plotted at each x-coordinate is the cumulative sum of fixations up to that time point, normalized by the number of fixations at that time point. (**c**) Social information intake rate *E*_*n*_ as a function of time in patch across travel times (1.0–7.0s). The black line indicates the maximum social value intake rate per travel time.

Next, we simulated optimal social information foraging by fitting individual monkey’s image-looking behavior to the MVT-based model (Eq. (3); Fig. 4a–c). Importantly, patch-leaving behaviors were modeled exclusively based on the duration of image-viewing. Therefore, the simulated optimal function does not take into account the influence of the juice reward delivered upon patch selection, which was consistent across all patch types and travel times. However, because the juice-maximizing strategy is to leave each patch immediately for the next patch’s juice reward, the juice reward likely influenced the social information gathering strategy to some extent. Comparing the simulated optimal social information foraging with the observed behavior in all three environment types indeed suggested a competitive influence of the optimal juice foraging strategy; the slope of the observed behavior in all environments indicated less sensitivity of patch-residence time to travel time (i.e., flatter slopes) than was predicted by the optimal social information foraging strategy (Fig. 3).

### Model Comparison

**Figure 5:**
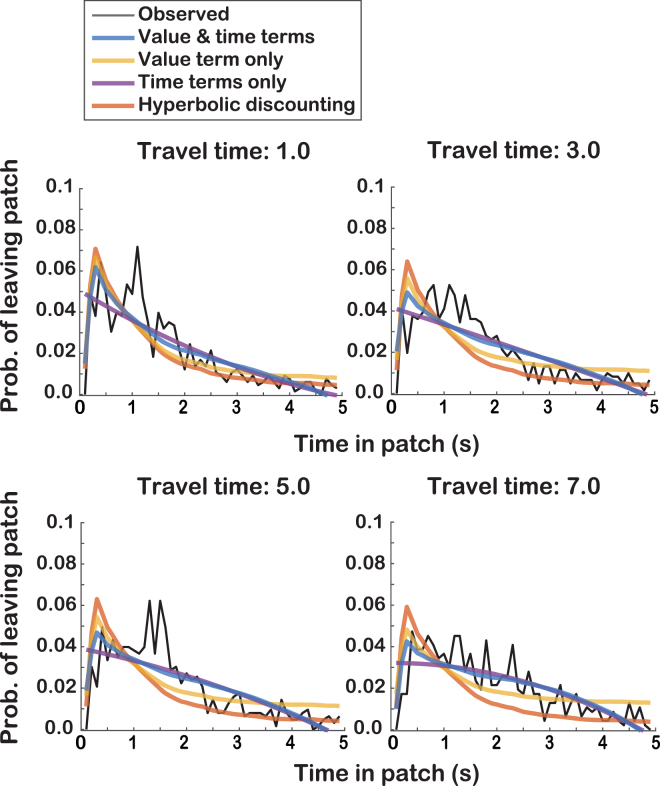
Fit comparison of simulations based on four patch-leaving models to observed social information foraging behavior. Probability of leaving the patch over time, comparing observed behavior (black) with simulations using (1) the adapted MVT model which included terms for social value *r* and time *t*, (2) a model with only a social value term (i.e., model 1 with time terms excluded), a model with only time terms (i.e., model 1 with value term excluded), and hyperbolic delay-discounting model for each travel time (see Methods).

**Figure 6:**
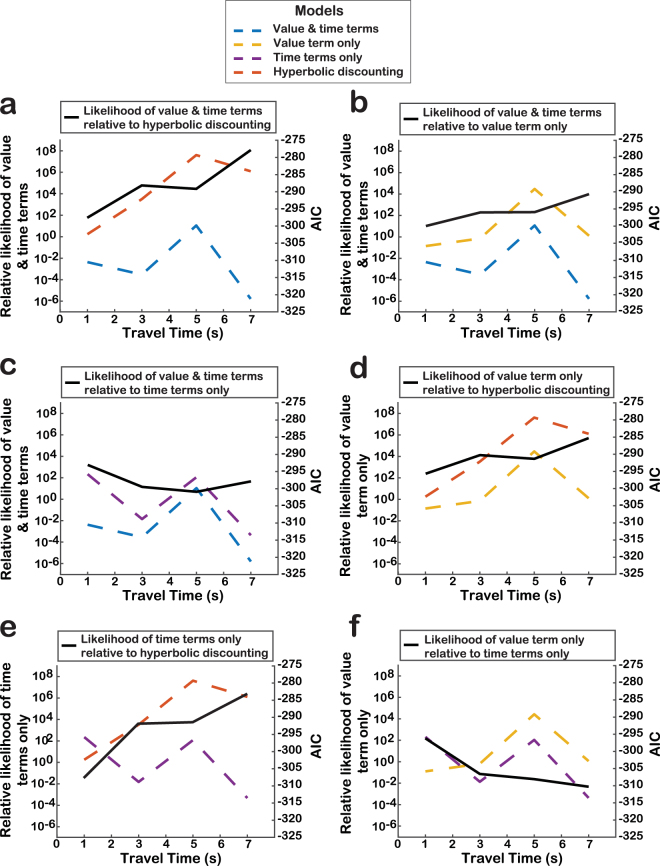
Akaike information criterion (AIC) and relative likelihoods of four patch-leaving models across travel times. Relative likelihood of model 1 (M1) versus model 2 (M2) was calculated as the ratio (Akaike weight_M1_)/(Akaike weight_M2_). Positive relative likelihood values indicate that M1 outperformed M2, and negative values indicate that M2 outperformed M1. All pairwise comparisons of the four models are shown (**a**–**f**).

We compared four models of patch-leaving probability to assess the validity of using an MVT-based approach. Comparison of the models (Table 1) revealed that the MVT-based simulation that included both *r*(t) and *t* terms consistently provided the best fit to the observed data (Fig. 5). Across all travel times, AIC values from this model were lower than those from all other models (Table 1), and the relative likelihood of this model, calculated as the ratio of model weights, was greater than zero across all pairwise model comparisons (Fig. 6). These results indicate that social information foraging in this task is better explained by the MVT framework than a simple decline in subjective valuation over time, and depends upon both the instantaneous value intake and current time in patch.

**Table 1:** Fit comparison of four models of patch-leaving behavior to observed social information foraging behavior.

Model	travel (s)	LL	*K*	AIC	dAIC	*w*	RMSE
MVT-based with value and time terms	1.0	159.248	—	−310.496	0	0.898	0.009
	3.0	161.065	—	−314.130	0	0.929	0.009
	5.0	153.914	—	−299.829	0	0.828	0.010
	7.0	164.600	—	−321.200	0	0.979	0.008
Value term only	1.0	154.907	—	−305.814	0	0.086	0.009
	3.0	153.800	—	−303.600	0	0.005	0.009
	5.0	146.586	—	−289.172	0	0.004	0.010
	7.0	153.404	—	−302.808	0	9.93 × 10^−5^	0.008
Time terms only	1.0	150.884	—	−295.768	6.587	0.001	0.009
	3.0	157.420	—	−308.841	0	0.066	0.009
	5.0	151.322	—	−296.644	0	0.168	0.010
	7.0	159.768	—	−313.536	0	0.021	0.008
Hyperbolic discounting	1.0	153.200	13.131	−302.356	8.140	0.015	0.011
	3.0	148.100	4.879	−292.140	21.990	1.56 × 10^−5^	0.012
	5.0	141.700	2.973	−279.308	20.521	2.90 × 10^−5^	0.013
	7.0	144.000	2.273	−284.027	37.173	8.29 × 10^−9^	0.013

Log likelihood (LL), number of free parameters (*K*), Akaike information criterion (AIC), delta AIC (dAIC), Akaike weights (*w*), and root mean square error (RMSE) were calculated for each level of travel time (travel). Value term refers to the current social value of the patch *r*(t), and time terms refers to *t* and *t* ^2^, where *t* is the current time in the patch.

## Discussion

The results support that social information foraging follows the principles underlying MVT. Specifically, we found support for our first hypothesis; on average monkeys stayed longer in patches when there was a higher time cost to traveling to the next patch. Furthermore, monkeys’ behavioral results could be approximated by MVT, though our simulation of optimal social information foraging under MVT tended to overestimate observed patch-residence time (Fig. 3a–c). This is likely a result of a competitive juice-maximizing strategy. Additionally, the results support our second hypothesis; across levels of travel time, MVT provided a better fit to the observed behavior than delay discounting. Thus, the canonical principle of MVT also applies to foraging for a social resource.

Monkeys assign different values to specific social resources, such as faces of dominant versus subordinate animals^16^. All monkeys in the current study displayed a strong preference to forage for social information from a specific category of facial expression. Monkeys preferred negative over non-negative expressions (similar to humans^21^), and social over nonsocial images. Dot probe task studies have shown that humans and bonobos are biased towards emotional images. Interestingly, humans are biased towards seeking information from threatening or aggressive images, whereas bonobos are biased towards sexual and affiliative images^21^, differences that likely reflect the social contexts in which these species live. Given the highly despotic social structure of rhesus macaques and the aggression that characterizes their social interactions^22^, prioritizing attention to negative-valence social cues over neutral or affiliative cues may be critical to survival (i.e., negative-valence cues have higher informational value). Curiously, despite exhibiting a preference for selecting negative over non-negative patches, monkeys’ looking times at negative and non-negative images were not statistically different. There are a number of potential explanations, the most likely of which are that (1) monkeys may harvest critical social information more quickly from negative-valence images since such images often require more rapid behavioral responses for survival, and/or (2) although negative-valence social cues have higher informational value, looking at these images is more aversive than looking at non-negative social images.

There is an important limitation to the behavioral preference data related to the grouping of different emotional expressions into two categories in the emotional valence environments. Though open-mouth threat and fear grimace have been categorized in previous work as negative-valence expressions as a grouping, ethologically, they represent distinct emotions and are used in different contexts^23^. Similarly, though lip smack^24,25^ and coo^23^ have been categorized as approach signals as a grouping, they are also often used in different contexts. Thus, future work can expand upon the preference information presented here by implementing a one-to-one mapping of stimulus and emotional expression category, rather than defining the category with respect to the perceived valence.

In all environments tested, monkeys’ behavior was consistent with the predictions of MVT in that patch-residence times increased with increasing travel time. The MVT-based model including *r*(t) and *t* terms outperformed the hyperbolic discounting model in describing social information foraging, suggesting that a subjective decline in valuation over time in the patch is not sufficient to describe the observed behavior. The model comparison also demonstrated that patch-leaving decisions depended upon both instantaneous value intake and current time in patch, as the model including both terms outperformed the models including *r*(t) only and *t* only (Table 1). Thus, our data provide evidence that monkeys use the same strategy to seek out social information from conspecific faces as they use to seek out juice^2^. Although previous work on nonsocial information seeking^9–11^ has shown that foraging for intangible nonsocial resources (e.g., searching the internet) also follows MVT principles, it remained unclear whether MVT also applies when individuals forage for more abstract and subjectively-derived information from other individuals. Our findings indicate that MVT applies to social information foraging.

The significant positive slope of the relationship between travel time and patch-residence time and the non-minimum patch-residence time even at the shortest travel time indicate a strategy for social information maximization (Fig. 3). However, a comparison of the monkeys’ actual behavior and the simulated optimal foraging model revealed that our MVT-based tended to overestimate time in patch (Fig. 3). In many applications of MVT (as well as other foraging models^26^), though the overall pattern (i.e., positive slope) of the model matches the observed behavior, quantitatively, the predictions show a systematic bias (e.g., refs^3,27–29^). In this case, the small effect size (i.e., shallow slope of the relationship between travel time and time in patch) was expected based on our MVT simulation. Because looking behavior begins declining within the first second in the patch and is essentially zero after 5 s in the patch, the additional social value extracted from an image by staying in the patch is low. The shallow slope was likely exaggerated by the influence of the constant-size juice reward that monkeys received after selecting each patch to sustain motivation. Shorter time in patch may suggest monkeys’ desire to return to the patch environment sooner to select a new patch and thus receive another juice drop. To maximize juice reward intake rate, subjects should immediately leave all patches while ignoring the travel time, and therefore patch-residence time across all travel times would be constant at the minimum time required to leave the patch (see Methods). Furthermore, our MVT simulation may only partially explain the behavior, as it derives social value solely based on looking behavior. Further knowledge on how to best estimate the value of social information will refine the MVT parameters and therefore the comparison between MVT and the observed behavior.

It is important to note that the social task described in this paper may be an example of a larger class of visual search problems that follow the same optimality principle. Future work might conceive a task that investigates social information foraging without relying on a visual search design. A task involving more complex social information would also elucidate whether there is a unique effect of social information above and beyond visual search as a general category. Because subjects were able to harvest critical social information so quickly in this task, it is difficult to assess this with the current dataset.

Our results extend the explanatory power of MVT in intangible information foraging to the social domain, providing novel evidence that animals use a similar strategy to enhance social resource intake as they use to maximize primary resource intake^2^. The demonstration that social information altered pure juice maximization suggests that individuals may balance competing strategies for optimizing values of social and nonsocial resources. The degree of symmetry in balancing foraging strategies across multiple resources likely depends on the value distributions across the social and primary resources available. Individuals may adaptively adjust the relative weight of primary versus social resources depending upon a variety of factors, including environmental conditions and availability of potential mates and social partners. Future work can help clarify this contingency by systematically manipulating the resource ratios between primary and social resources. Finally, phenotypic differences among individuals may also contribute to differences in social information use^30^ and predictably relate to social competency.

## Methods

### Animals and General Procedures

Three male rhesus macaques, Monkeys L, J, and K (16.5, 8.0, and 5.6 kg; aged 9, 5 and 4 years), participated. Monkeys resided in a colony containing two dyads (all male) and two triads (one all female, one all male), and had visual access to all monkeys in the room. Monkey L was dominant over Monkey K and the other juvenile male in his triad, Monkey K was subordinate to Monkey L and of equal rank with the other juvenile male in his triad, and Monkey J was subordinate to the other male in his dyad. Despite dominance differences, all three monkeys showed similar effects (Fig. 2a).

Animals received a surgically implanted headpost (Crist Instruments or GreyMatter Research) for restraining their heads while tracking eye positions at 1,000 Hz (EyeLink, SR Research). All procedures were conducted in accordance with the National Institutes of Health guidelines and the Public Health Service’s Guide for the Care and Use of Laboratory Animals, and with approval from the Yale University Institutional Animal Care and Use Committee.

### Testing Procedures

#### Social Information Foraging Task

Monkeys explored three block types representing three different virtual foraging environments. In each block subjects were presented with two different target types. Target color cued the category of the image that would be presented upon selecting the target. During image display, the subject could choose to begin a new trial (i.e., to return to target selection) by making a saccade to a rectangular target (“the travel bar”) presented alongside the image. The travel bar was oriented vertically and its height indicated the duration of the delay the subject must wait through from the time the travel bar was selected to the time at which the targets would be presented again. In foraging theory terms, our blocks were foraging “environments”, the targets were “patches”, and the travel bar indicated “travel time” to the next patch. Each patch contained one image of a novel conspecific face that was randomly selected from the bank of task stimuli (see below) with replacement.

The foraging environments included (1) *a color-cued emotional valence environment* with negative and non-negative (cued by red and blue targets, respectively) valence facial expressions (2), a *fractal-cued emotional valence environment* that also presented negative and non-negative facial expressions, but the targets were two black-and-white fractal patterns to ensure that preferences were based on image valence rather than target color, and (3) a *color-cued social vs nonsocial environment* in which monkeys chose between neutral-expression and scrambled conspecific faces (cued by yellow and purple targets, respectively) to ensure their baseline preferences for social images (Fig. 1b).

Each environment contained eight total patches [targets], including four of each patch type (Fig. 1a). Patches were randomly distributed with 22.5° spacing around an imaginary circle with an eccentric of 4.90° of visual angle. When the monkey selected a patch by making an eye movement to the patch location, a juice reward of fixed amount was delivered via solenoid to sustain task engagement, followed by a delay (“handling time”, 400 ms). Critically, the juice reward was given immediately before entering a patch and had no bearing on patch preferences, as the volume was consistent across patch types. After handling time elapsed, the image associated with the selected patch was displayed in the center of a black screen and monkeys had up to 15 s of free-viewing. During this period, the travel bar was displayed to one side of the image. The height of the bar indicated “travel time” (the longer the travel time, the taller the travel bar), which varied randomly from 1.0 s to 7.0 s in 2.0-s intervals for Monkeys L and K and from 0.5 to 5.0 s in 0.5-s intervals for Monkey J. Monkeys could leave the current patch at any time during the free-viewing period by fixating on the travel bar for 200 ms, at which time the image disappeared and the travel bar began shrinking. If the travel bar had not been selected by the end of the 15-s free-viewing period, the image disappeared and travel bar began shrinking automatically (these trials were not included in the MVT analyses). The rate of shrinking was timed such that the travel bar was completely shrunk when the travel time had expired. Then, the task returned to the current environment where the remaining (unvisited) patches were displayed in their original locations. After 70.0 s within the environment had elapsed or after all patches in the environment had been selected, the task entered an inter-environment interval of 5.0 s, after which the monkey entered a new environment with eight new patches.

All monkeys completed all three environment types. Testing days consisted of 1- to 1.5-hour sessions per environment type with up to two environment types per day (the order was randomized across days). On each day, monkeys visited 303 ± 12 [mean ± standard error] patches. The task was implemented in Matlab (Mathworks) with Psychophysics Toolbox^31–33^ and Eyelink Toolbox^34^.

#### Task Stimuli

The stimuli included 199 negative and 199 non-negative facial expression images for both emotional valence environments (*color-cued* and *fractal-cued*), as well as 100 social (neutral expression), and 100 nonsocial (scrambled versions of the neutral images) for the *color-cued social vs nonsocial environment* (images from ref.^24^). Each category contained photographs of a minimum of 25 different individuals, and all images were of unfamiliar monkeys (i.e., monkeys that do not reside in the same colony) to simulate males’ dispersal to new social groups.

When a monkey selected a patch, one image was randomly drawn from the bank of task stimuli for the corresponding category (i.e., negative or non-negative; social or nonsocial) with randomized replacement. In both emotional valence environments, open-mouth threat and fear grimace were grouped as negative-valence expressions^23^, and lip smack^24,25^, coo^23^, and neutral faces were grouped as non-negative expressions (Fig. 1b). Open-mouth threat is typically given by dominants to subordinates as an aggressive approach signal^23^. Fear grimace is a negative withdraw signal typically given by subordinates to dominants^23^. Coo is an affiliative approach signal typically given during grooming interactions, eating, or affiliative approaches^23^, and lip smack is an approach signal typically given by subordinates to dominants or by individuals engaged in grooming^23^, which is often followed by affiliation^25^.

### Data Analysis

Contrast ratios (CR) were calculated daily to assess the direction and strength of patch choice preferences of each monkey within each environment.

1 $$CR=\frac{({F}_{A}-{F}_{B})}{({F}_{A}+{F}_{B})}$$

For both emotional valence environments, *F*_*A*_ and *F*_*B*_ were the numbers of non-negative and negative images selected, respectively. For the *color-cued social vs nonsocial environment*, *F*_*A*_ and *F*_*B*_ were the numbers of social and scrambled images selected, respectively. The ratios ranged from −1 to 1, with 0 indicating indifference. Preferences within each environment were assessed using a student *t* test.

We examined the sequential order of patch selection within each *color-cued emotional valence* environment (i.e., within each set of eight patches) as the cumulative proportion of negative or non-negative patches selected out of the total patches for each patch type (starting with four negative and four non-negative) as a function of choice number (up to eight choices). We analyzed these data using Pearson’s chi-squared test. Average looking times for negative versus non-negative images were calculated based on looking within the bounds of the face, and were evaluated using a Wilcoxon signed-rank test, as looking times were not normally distributed.

### MVT simulation

We applied the MVT by evaluating the behavior according to the MVT equation,

2 $${E}_{n}\,=\frac{{\sum }^{}{P}_{i}\,\times \,{g}_{i}({T}_{i})-t\times \,{E}_{T}}{t+{\sum }^{}{P}_{i}\times {T}_{i}}$$

where *E*_*n*_ is net energy intake rate (in this case, the net social value intake rate), *P*_*i*_ is the proportion of patches of a given type, *g*_*i*_ is the amount of energy gained corrected for the energetic cost of searching (the social value intake function for a given image over time in the patch), *T*_*i*_ is time spent hunting (time in patch), *t* is travel time (time from selecting the travel bar to returning to fixation), and *E*_*T*_ is the energy cost per unit time during traveling^6^.

We made the same assumptions made by a previous study investigating nonsocial resource foraging in rhesus macaques^2^: that the (1) cost of searching was zero, (2) energy cost per unit time during travel was zero, (3) patches of a given type were identical, and (4) handling time was constant across patches (400 ms). We made the simplifying assumption that the information capture rate function *g(t)* is the same for all patches in a given environment, i.e., there is effectively one patch type in each environment in our task. Thus, the cost of search, travel, handling time, and rate of information capture remained constant, but energy (i.e., social value) gain per encounter varied among patches and declined as a patch was exploited. Under these assumptions, net energy, or social value, intake rate of a patch was defined as

3 $${E}_{n}=\frac{g(T)}{t+T}$$

The value of the patch decreases over time spent in the patch because the social value of the image diminishes with time as social information is harvested and novelty declines. Maximal value intake rate is achieved when the intake rate within a patch is equal to the average intake rate for the environment^4^.

We simulated expected behavioral results according to optimal social information foraging using our MVT-based model. We used the looking behavior of monkeys while they were within patches (Fig. 4a) to derive the value of social images (i.e., the duration of total fixations on the image in the patch; see below). For each trial, we recorded whether or not the monkey was fixating on the image (binary of 1 or 0) over the course of the 15-s period. We then summed these values at each time point across all trials and binned the data by 100-ms to produce a cumulative distribution function (Fig. 4b). Given the large sample size for number of fixations (*n* > 7 × 10^6^), we let the MVT capture rate function *g(t)* be directly proportional to the empirically observed cumulative distribution function.

With *g(T)*, the social value intake rate *E*_*n*_ was quantified as a function of time in patch across travel times (Eq. (3); Fig. 4c). We then identified the maximum social value intake rate for each travel time, representing optimal time in patch as a function of travel time, a prediction of how monkeys should behave to optimize social information foraging according to MVT. We also simulated expected behavioral results if monkeys optimize juice - a primary, nonsocial resource. In our task, monkeys could maximize juice intake rate by leaving each patch immediately, regardless of travel time and without viewing the image. Thus, patch-residence time under optimal juice foraging should ignore the travel time and therefore is theoretically the minimum fixation required to select the travel bar, 200 ms (when not considering any nonspecific time required for eye movement) across all travel time values (i.e., slope of zero). Because the animals could be looking at any position inside or outside the screen (outside the limit of eye tracking) when the image was first revealed, calculating the nonspecific eye movement related times becomes problematic. For the purpose of displaying the theoretical optimal juice and social foraging curves, we added an additional 250 ms to both curves as a minimum estimate of saccade reaction time and movement time.

### Test of MVT

To determine whether monkeys’ behavior was consistent with the predicted behavior, we assessed patch-residence time as a function of travel time. MVT predicts a positive relationship between patch-residence time and travel time, which we tested for using a one-tailed Kendall’s correlation test. Because the majority of image-viewing behavior (Monkey L: 97.7%, J: 92.1%, K: 98.1%) and patch-leaving decisions (Monkey L: 88.5%, J: 86.3%, K: 78.4%) occurred within the first 5 s of the image viewing period, we focused on patch-residence times falling within this period (0–5 s), as the later period (5–15 s) was more likely to be biased by distraction or disengagement from the task. Indeed, the looking behavior function indicates categorically different behaviors in these periods (Fig. 4a).

### Model Comparison

We compared four models of patch-leaving behavior to determine whether a different model may describe the data better than our MVT-based model. Within each environment, the observed data were collapsed across monkeys and patch-leaving models were fitted separately for each level of travel time shared by at least two of the three monkeys (travel times 1, 3, 5, and 7 s).

To model patch-leaving behavior, we calculated the probability of leaving the patch in a given time segment over the first 5 s of the image-viewing period. We binned patch-residence times into non-overlapping 100-ms bins, and calculated the proportion of patch-residence times within each bin. We operationalized the value of the social information as the frequency of looks to the image, binned into non-overlapping 100-ms bins (i.e., within each bin the amount of time [ms] spent looking at the image was summed over all trials).

Charnov’s original MVT describes only average patch-leaving behavior^4^. To describe trial-to-trial variability in patch-leaving behavior, we adapted Hayden’s model^2^, which is based on MVT but modified to describe trial-by-trial behavior. Our ad hoc MVT-based model for patch-leaving behavior in the context of this task is therefore,

4 $${P}_{leave}={b}_{0}+{b}_{1}\times r+{b}_{2}\times t+{b}_{3}\times {t}^{2}$$

where *b*_0_ is the intercept, *r* is the current social value (i.e., looking behavior in a given bin *x*), and *t* is the current time in the patch. We refer to this model as the “value and time terms model”, which refers to the inclusion of both the social value term *r*(t) as well as time terms *t* and *t* ^2^.

We compare this to a purely time-based model,

5 $${P}_{leave}={b}_{0}+{b}_{1}\times t+{b}_{2}\times {t}^{2}$$

where the value term *r* is omitted. We refer to this as the “time terms only” model. If the value term included in equation (4) improves the model fit relative to equation (5), this would demonstrate that patch-leaving behavior is sensitive to instantaneous rate of social value intake, consistent with MVT.

A third variation of our MVT model is one that is independent of time,

6 $${P}_{leave}={b}_{0}+{b}_{1}\times r$$

where both time terms *t* and *t* ^2^ are omitted. We refer to this as the “value term only” model. If the exclusion of the time terms improves the model fit relative to equation (4), this would be a more parsimonious approximation of patch-leaving behavior.

Another alternative is that patch-leaving behavior is solely tied to the ‘subjective value’ of the remaining social information. In this case, patch-leaving decisions should be better captured by a standard hyperbolic delay-discounting function, in which the ‘value’ of the resource in the patch is inversely scaled by the delay required to receive it,

7 $${P}_{leave}=\frac{r}{1+k\times t}$$

where *r* is the declining social information value, *k* is the discount parameter fitted using a maximum likelihood method, and *t* is the time to next reward (i.e., time delay to extracting the next additional unit of social value). Thus, for patch-leaving decisions, *t* is the travel time to the next patch. We refer to this as the “hyperbolic discounting” model.

To compare the four models, we calculated log likelihoods (*LL*) and obtained Akaike information criterion (AIC) values using the equation,

8 $$\mathrm{AIC}\,=(-2\times LL)+(2\times k)$$

where *k* is the number of free parameters in the model. We then transformed the AIC values into Akaike weights, *w*, which can be interpreted as the conditional probabilities for each model^28^. Each *w*_*i*_ is defined as

9 $${w}_{i}=\frac{{e}^{-(\frac{1}{2}){\ast {\rm{\Delta }}}_{i}}}{{{\sum }^{}}_{i}{e}^{-(\frac{1}{2}){\ast {\rm{\Delta }}}_{i}}}$$

We conducted pairwise comparisons of the model fits by dividing the Akaike weight of each model by the corresponding weight of each other model across travel times^35,36^.

### Data Availability

The datasets, analysis code and other relevant materials are available through https://github.com/changlabneuro/social_foraging.
